# Avascular Necrosis of the Hip: A Post COVID-19 Sequela

**DOI:** 10.7759/cureus.29976

**Published:** 2022-10-06

**Authors:** Tyler J Kingma, Virginia Hoch, Chelsey Johnson, Bilal Chaudhry

**Affiliations:** 1 Internal Medicine, Edward Via College of Osteopathic Medicine, Spartanburg, USA; 2 Internal Medicine-Pediatrics, Christiana Care Health System, Newark, USA; 3 Internal Medicine, Christiana Care Health System, Newark, USA

**Keywords:** hip joint pain, sars-cov-2 (severe acute respiratory syndrome corona virus 2), avascular femoral head necrosis, avascular necrosis (avn), bilateral femoral neck fracture, osteomyelitis, covid-19

## Abstract

A 60-year-old African American male presented to the hospital with seven months of progressively worsening left anterior hip pain with no known trauma. Two months after the pain onset, he underwent an x-ray of the pelvis with the lateral left hip, revealing dystrophic soft tissue calcification adjacent to the superolateral left acetabulum. Pain at this time was attributed to presumed sciatica vs arthritis. The patient underwent multimodal treatment for his pain without relief. In the month prior to the presentation, the patient also developed right hip pain. He then underwent a bilateral hip x-ray, revealing left femoral neck lucency suspicious for a nondisplaced fracture. CT pelvis was ordered at this time for further evaluation and demonstrated bilateral subcapital hip fractures. He was subsequently discharged from the emergency department with pending laboratory work and plans for close outpatient orthopedic surgery follow-up. The following day, the patient was instructed to return to the hospital due to an elevated erythrocyte sedimentation rate of 39 mm/hr and C-reactive protein of 41.6 mg/L. Subsequent MRI pelvis revealed bilateral subcapital femoral neck fractures with avascular necrosis (AVN) requiring surgical intervention with bilateral hip arthroplasty. Our patient underwent an extensive workup with no evidence of traditional risk factors for osteonecrosis, osteopenia, or other bone diseases. A pertinent finding in the patient’s history was an admission for severe SARS-CoV-2 (COVID-19) infection 10 months prior. ‘Long COVID’ is a complex illness that has been shown to affect intravascular blood flow, and likely contributed to the development of bilateral hip AVN in our patient. Given this novel presentation, it is crucial that AVN be considered early in evaluating anterior hip pain for patients with a history of COVID-19 infection in order to avoid severe consequences such as femoral neck fractures.

## Introduction

We present a case of avascular necrosis (AVN) resulting in bilateral femoral neck fractures as a sequela of COVID-19 infection. Both COVID-19 infection and avascular necrosis are relatively common, however, there has been minimal published data regarding a relationship between the two. Although many questions still remain about COVID-19, there have been many studies and reports showing a profound prothrombotic state on both a micro and macrovascular level in those with recent infections. However, less information is available in regard to COVID-19 being a catalyst for avascular necrosis development. While there are variable triggers of atraumatic AVN, the most common causes are alcohol, steroid use, and sickle cell disease. Despite the diverse pathology of these different etiologies, a combination of vascular impairment, altered bone-cell physiology, and genetics appear to contribute to common pathophysiology [1]. It is still unclear if COVID-19 infection induces one or all of these pathophysiological changes, but this case would suggest that COVID-19 should also be considered as a cause of atraumatic AVN. 

## Case presentation

A 60-year-old African American male with a past medical history significant for alcohol use disorder (last drink 10 months prior), hypertension, hyperlipidemia, coronary artery disease, and COVID-19 infection ten months prior requiring admission to the intensive care unit, presented to the hospital with seven months of progressively worsening left anterior hip pain with no known trauma. Two months after pain onset, he underwent an x-ray of the pelvis with the lateral left hip, revealing dystrophic soft tissue calcification adjacent to the superolateral left acetabulum. Pain at this time was attributed to presumed sciatica vs arthritis. The patient underwent treatment for his pain with multiple rounds of physical therapy along with medications such as gabapentin, acetaminophen, cyclobenzaprine, and a four-day course of prednisone. In the month prior to the presentation, the patient also developed right hip pain. He then underwent a bilateral hip x-ray, revealing an irregular linear lucency through the junction between the head and neck of the left femur suspicious for a nondisplaced fracture. CT pelvis was ordered at this time for further evaluation and demonstrated bilateral subcapital hip fractures. He was subsequently discharged from the emergency department with pending laboratory work and plans for close outpatient orthopedic surgery follow-up. The following day, the patient was instructed to return to the hospital due to an elevated erythrocyte sedimentation rate of 39 and a C-reactive protein of 41.6 (reference ranges below). He was afebrile, with vital signs otherwise unremarkable. His physical exam was remarkable only for the reproduction of pain with internal and external rotation of the bilateral hips. He had an intact range of motion of the lower extremities and lacked focal tenderness to palpation.

**Figure 1:**
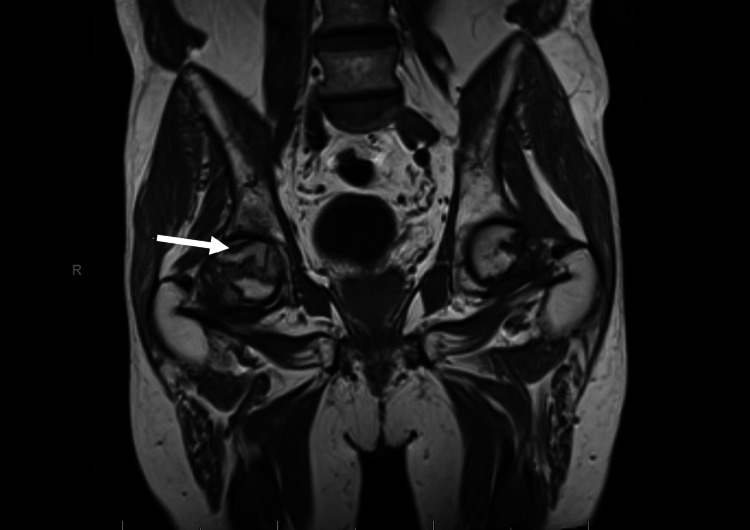
MRI pelvis showing marked marrow edema and enhancement in the right femoral neck

Initial hospital labs (Table 1) revealed non-specific elevation of erythrocyte sedimentation rate and C-reactive protein. White blood cell count, calcium, vitamin D, thyroid stimulating hormone (TSH), and parathyroid hormone (PTH) were within normal limits. Hemoglobin electrophoresis (Table 2) and iron studies were performed due to microcytic anemia discovered during his admission, revealing 97.3% hemoglobin A and an iron level of 42mcg/dL. Serum protein electrophoresis (Table 2) was performed to evaluate for possible multiple myeloma in the setting of anemia and bilateral femoral neck fractures and was found to have no evidence of a monoclonal gammopathy. CT pelvis without contrast as mentioned above revealed age-indeterminate mildly impacted left femoral neck subcapital fracture and fracture of the right subcapital femoral neck. Subsequent MRI pelvis revealed alteration in the femoral heads bilaterally, consistent with sequela of avascular necrosis. It also showed nondisplaced subcapital fractures involving the femoral neck bilaterally with marked associated marrow edema and enhancement seen in the right femoral neck as shown in Figure 1.

**Table 1:** Summary of Initial Laboratory Findings

Laboratory finding	Value	Reference range
White blood cell (10^9^/L)	10.4	4.5-11
Hemoglobin (g/dL)	13.7	13.2-16.6
Hematocrit (%)	42.3	41-50
Mean Corpuscular Volume (fL)	71.2	80-100
Platelets (10^9^/L)	249	150-400
Sodium (mmol/L)	132	135-145
Potassium (mmol/L)	3.7	3.5-5
Chloride (mmol/L)	99	95-105
Total CO2 (mmol/L)	23	18-22
Creatinine (mg/dL)	1.02	0.8-1.3
Blood urea nitrogen (mg/dL)	17	8-21
Aspartate aminotransferase (U/L)	21	5-30
Alanine transaminase (U/L)	36	5-30
Albumin (g/L)	4.1	3.5-5
Erythrocyte Sedimentation Rate (mm/hr)	39	<22
C-Reactive Protein (mg/L)	41.6	<5
Vitamin D 25-OH (ng/mL)	34	5-75
Thyroid Stimulating Hormone (mIU/L)	2.44	0.5-5
Parathyroid Hormone Intact (pg/mL)	21	10-55
Iron (mcg/dL)	42	60-150
Ferritin (ng/mL)	189	40-200
Total iron-binding capacity (mcg/dL)	356	300-360
Transferrin saturation (%)	12	20-50
Blood cultures	Negative	

**Table 2:** Hemoglobin Electrophoresis and Serum Protein Electrophoresis (SPEP)

Hemoglobin Electrophoresis		Reference range
Hemoglobin A (%)	97.3	95-98
Hemoglobin A2 (%)	2.3	2-3
Hemoglobin F (%)	0.4	0.8-2
Hemoglobin S (%)	0.0	Absent
Hemoglobin C (%)	0.0	Absent
Serum Protein Electrophoresis		
Serum total protein (g/dL)	6.7	6.4-8.3
PEP Albumin (g/dL)	3.4	3.5-5.0
PEP Alpha 1 (g/dL)	0.4	0.1-0.3
PEP Alpha 2 (g/dL)	1.0	0.6-1.0
PEP Beta (g/dL)	0.9	0.7-1.2
PEP Gamma (g/dL)	1.0	0.7-1.6

The patient underwent successful surgical intervention with bilateral total hip arthroplasty during his admission, which he tolerated without complication. The postoperative course was complicated by down-trending hemoglobin levels that stabilized after one unit of blood prior to discharge. Tissue cultures of the femoral head were negative for any growth. He began participating in physical therapy on postoperative day one and was ultimately discharged home on postoperative day four with home physical therapy.

## Discussion

Atraumatic AVN of the hip is described as a lack of blood supply to the proximal femur. Approximately 80% of cases of AVN are a result of chronic corticosteroid use or chronic alcohol use [2]. Though this patient has a history of both corticosteroid and alcohol use, there is a lack of consensus in the literature about the dose, duration, and timing of use that can lead to AVN. This is highlighted in a meta-analysis evaluating 57 studies performed by Mont et al. evaluating high-dose corticosteroid use and the risk of osteonecrosis. Evaluation of various inflammatory and autoimmune diseases in this analysis revealed a wide variation of mean cumulative corticosteroid dosing during the duration of treatment, ranging from 2,000 mg to greater than 20,000 mg over the course of one to ninety-six months [3]. A limit of the meta-analysis, however, is that there is no mention of whether the corticosteroid values are corrected to a prednisone equivalent. The total dose of prednisone equivalent our patient received was 1,327.5 mg over the course of one month, which is less than the dose found to be significantly associated with the development of AVN. Therefore, his presentation does not suggest that corticosteroids were the primary cause of his bilateral hip AVN.

Chronic alcohol use is also well-documented as a cause of avascular necrosis in a dose-response manner across the literature [4]. However, there is little to no data on the incidence of avascular necrosis in patients with a history of chronic alcohol use but who are no longer consuming alcohol. Our patient quit drinking alcohol 10 months prior to this admission in the setting of his COVID-19 infection, making alcohol use also less likely to be the primary cause of AVN.

While no laboratory workup can definitively suggest or confirm the presence of avascular necrosis, our patient underwent extensive workup to assess for other risk factors for osteonecrosis, osteopenia, or other bone diseases that may have made him prone to atraumatic fractures. Sickle cell disease was ruled out by hemoglobin electrophoresis. Calcium, 25-hydroxy vitamin D, TSH, PTH, and serum protein electrophoresis were not suggestive of accelerated bone resorption. In addition, there was no evidence to suggest a malignant process on imaging nor based on history and exam, as the patient denied recent weight changes or constitutional symptoms.

We believe our patient experienced bilateral femoral head AVN as a complication of COVID-19.

Acute COVID-19 infection can manifest in many ways from nominal symptoms to catastrophic organ failure. ‘Long COVID’ is a complex, multifactorial illness that has been shown to have some effect on nearly all organ systems, including creating a profound prothrombotic state on both a micro and macrovascular level [5]. Complications from this prothrombotic state have included such issues as renal failure, deep venous thrombosis, pulmonary embolism, myocardial infarctions, cerebrovascular accidents, and loss of digits/limbs [5]. It may be this prothrombic state that led to a veno-occlusive state in our patient. There are only a small number of case reports with similar findings of femoral AVN following a COVID-19 infection [6]. Only two of those showed bilateral AVN and none with such delayed presentation resulting in bilateral femoral neck fractures [7]. In our patient, it is likely that there was some degree of AVN present at the onset of symptoms, seven months prior to diagnosis. If diagnosed earlier, it is possible that he would not have progressed to bilateral subcapital femoral neck fractures requiring bilateral total hip arthroplasty. This is a novel presentation of AVN that should now be considered in a patient with new-onset hip pain in the setting of a recent COVID-19 infection, with a low threshold to evaluate with MRI imaging.

## Conclusions

‘Long COVID’ is a complex illness that has been shown to affect intravascular blood flow causing a hypercoagulable state, and likely contributed to the development of bilateral hip AVN in our patient. Given this novel presentation, it is crucial that AVN be considered early in evaluating anterior hip pain for patients with a history of COVID-19 infection in order to avoid severe consequences such as femoral neck fractures.

## References

[1] Liu YF, Chen WM, Lin YF (2005). Type II collagen gene variants and inherited osteonecrosis of the femoral head. N Engl J Med.

[2] Petek D, Hannouche D, Suva D (2019). Osteonecrosis of the femoral head: pathophysiology and current concepts of treatment. EFORT Open Rev.

[3] Mont MA, Pivec R, Banerjee S, Issa K, Elmallah RK, Jones LC (2015). High-dose corticosteroid use and risk of hip osteonecrosis: meta-analysis and systematic literature review. J Arthroplasty.

[4] Yoon BH, Kim TY, Shin IS, Lee HY, Lee YJ, Koo KH (2017). Alcohol intake and the risk of osteonecrosis of the femoral head in Japanese populations: a dose-response meta-analysis of case-control studies. Clin Rheumatol.

[5] Manolis AS, Manolis TA, Manolis AA, Papatheou D, Melita H (2021). COVID-19 infection: viral macro- and micro-vascular coagulopathy and thromboembolism/prophylactic and therapeutic management. J Cardiovasc Pharmacol Ther.

[6] Sulewski A, Sieroń D, Szyluk K, Dąbrowski M, Kubaszewski Ł, Lukoszek D, Christe A (2021). Avascular necrosis bone complication after active covid-19 infection: preliminary results. Medicina (Kaunas).

[7] Agarwala SR, Vijayvargiya M, Pandey P (2021). Avascular necrosis as a part of 'long COVID-19'. BMJ Case Rep.

